# An innovative proposal for scientific alternative medical journals

**Published:** 2017

**Authors:** G Vithoulkas

**Affiliations:** * University of the Aegean, Greece

## Letter to the Editor

Contemporary homeopaths all over the world are witnesses to one of the strangest things to have ever occurred in our complex modern scientific society, namely, that our most prestigious homeopathic journals with an “impact factor” rarely, if ever, publish studies on cases treated and cured with homeopathy. Why is this so? [**1**]

Let us examine this question. It is a well-known fact in the international homeopathy community that every day, there are literally thousands of chronic sufferers treated successfully all over the world through the intervention of homeopathic remedies. All homeopaths have observed the occasional “miraculous cures” occurring in their own practice and in those of their colleagues. However, despite these remarkable “cures”, it is very strange that hardly any of these evidential cases appear in our homeopathy journals.

Homeopaths and patients know that millions of successful treatments occur all the time and all over the world. However, it appears that the editors of relevant journals are blissfully unaware of this fact. Their screening protocol is so effective that case studies are unable to pass even the most lackadaisical of peer reviewers. These “master” peer reviewers are usually grossly uninformed about real homeopathy and its rules and principles. Most of them are neither prescribers nor teachers of homeopathy! These “self-appointed” doyens of homeopathy guard the pillars of sound “scientific evidence” with such enthusiastic vigor that no evidence whatsoever is allowed to become public knowledge.

Yet, there is irrefutable evidence, that this planet is a veritable cornucopia of successfully treated homeopathic cases. The multitude of successes can be evidenced by the fact that homeopathy is practiced effectively in overpopulated countries, such as India, Pakistan, Brazil and other South American countries. Against such overwhelming evidence, it is truly remarkable that these so-called “scientific guardians” of our science manage to employ the most absurd excuses for not publishing studies on cured cases.

However, the only evidence that homeopathy can present to the scientific world at this moment are these thousands of cured cases. It is a waste of time, money, and energy to attempt to demonstrate the effectiveness of homeopathy through double blind trials.

Because of this neglect, the international “scientific” community, which has neither direct perception nor personal experience of the beneficial effects of homeopathy, is forced to repeat the same old mantra: “Where is the evidence? Show us the evidence!” Because of these gross omissions by the peer reviewers of the “scientific” homeopathy journals, the successes of homeopathy have remained hidden in the offices of hardworking homeopaths - and thus go largely ignored by the world’s medical authorities, governments, and the whole international scientific community.

Because of these tactics, the genius of the homeopathic system of medicine continues to be ignored at large, with the side effect being that millions of sick people, unaware of its existence, continue to suffer needlessly.

It should be added here that homeopathy, being an individualized system of medicine, could only present results on individual cases. Homeopathy is about individualization - not generalization. This treatment modality cannot produce a remedy that will cure cancer, asthma, multiple sclerosis, ulcerative colitis or any other chronic disease - but it has the potential of curing many of such cases, if treated correctly and individually with the patients indicated remedy. Therefore, simple questions that are usually asked by the “gnorant”, for example, “Can homeopathy cure cancer, multiple sclerosis, ulcerative colitis, etc.?” are invalid and cannot elicit a direct answer because the reality is that many such cases can be ameliorated significantly, and a number can be cured.

If they refuse to publish crucial evidence of well- managed homeopathic cases in the scientific homeopathy journals, where on earth can this palpable proof be presented such that all concerned can be made aware of, and judge for themselves, the merits of this important therapeutic modality?

I surmise that there are three possible reasons for this unfortunate state of affairs:

a. Either there is an organized effort to disallow the crucial evidence to surface, a theory that I personally do not believe, since there is no evidence for it

b. The “scientific” homeopathy journals are reluctant to present cured cases because they fear criticism, or

c. The thinking of the peer reviewers is so inexplicably complex and complicated that they find themselves rejecting a successful case even when the evidence is beyond any doubt.

Another disturbing point is that some homeopathy journals quite categorically state that they will not accept studies of cured cases!

I would propose another strategy. If these journals would choose to invite homeopathic doctors to report their cured cases and their failures, as well, then a huge body of important evidence could be amassed of what homeopathy can or cannot do.

Homeopathy is a dynamic system of medicine that has the potential for significant growth and helping to deal with many of the global health issues that exist today. However, we still need to solve many concerns and to address many unanswered questions.

Why, for instance, in a case of rheumatoid arthritis, one patient is cured with one or two remedies in a period of a few months, while another needs four or more remedies in a period of several years, even with careful prescribing? What are the parameters that define one or the other response?

Why, in one case, is the daily repetition of a high potency a false tactic with a negative outcome, while in another case, it is necessary and associated with positive results?

Why do low potencies act better in one case, while high potencies are best for another patient, even when they have the same pathology?

Why, in certain cases, do we have a strong initial aggravation, while in others, the effect goes smoothly and without aggravation?

Is the return of old symptoms a good omen for a lasting cure?

Do we understand what really occurs with this type of development in a case? Should such old symptoms be treated or left to resolve themselves? When should we expect the return of old symptoms? Does this occur in all cases?

What are the parameters that show that a remedy acts as a palliative and not as a curative agent? Which are the signs that a remedy has acted deeply and curatively, as opposed to acting by only disturbing the organism? [**2**,**3**]

I can mention hundreds of such questions, but the answers are not the work of a single individual but of an international group of good prescribers. Such an endeavor could be undertaken by a prestigious journal that has the means -financial and scientific - to perform such a task.

A journal could invite a selected number of good prescribers from all over the world as a start to this project and let them contribute to their honest experience and results, as well as their failures. The possibilities and limitations would soon be revealed.

In this way, homeopathy will become interesting and alive, and the readership will increase spectacularly.

For instance, due to technological advances, it is now possible to collate hundreds of gangrene cases from all over the world: seriously progressed cases where amputations were deemed necessary to show the world that these people can now walk on both legs again. The same is possible with vitiligo, where the effect is obvious. [**4**,**5**]

The fact is that in all such cases, it will be found that they are treated with different remedies and that a double-blind trial is therefore not applicable, or even when applied, a series of compromises would be necessary on different levels.

I personally have evidence of a video that in 1990, in front of three hundred doctors in Celle, Germany, where I was giving a seminar, I treated a case of a 72- year-old woman with advanced (diabetic) gangrene who had entered the nearby hospital for amputation of both legs at the level of her thighs. In three days, and while the seminar was in progress, blood flow was re-established in her legs after two days of treatment and the woman was discharged from the hospital after 10 days with both her legs intact. [**6**]

Ten years later, a letter from her daughter, who holds an MD and attended my course, confirmed that the old woman lived peacefully and was going to walk on her own on two feet for the next ten years. Without the intervention of homeopathy, this woman would have lived the last years of her life in a wheelchair.

There are literally hundreds of cases similar to this being successfully treated in such countries as India and Pakistan, where this pathology is prevalent. Evidence could be presented through photos, videos and other modern high-tech media.

Why should we suppress such significant tangible proof of the effectiveness of homeopathy at such a crucial time in the history of medicine? When, more than at any other time, do we need to clarify the confusion that has been created in matters of health?

By failing to publish cases, we hide the potentials of such an impressive therapeutic system.

Homeopathy is not able to cure all chronic diseases, especially if it has advanced beyond a certain point in its pathology. Conversely, homeopathy has the potential to successfully treat diseases that conventional medicine cannot cure or, in certain instances, cannot even palliate. Is it not the task of a serious homeopathic journal to make available its platform to discuss and explain these matters?

I admit that an argument against accepting cases is that it is possible that false or unreliable information could be provided. This risk could be minimized by preselecting a well-known group of good prescribers, who could be asked to submit their cases, at least in the first phase of such a radical change in the policy of the journals.

A platform for sending studies of cases could be constructed with guidelines to ensure reliability.

Another possibility could be a validation from a small body of local experts that could act as assessors. These experts may be based in each country and associated with the journal. [**7**,**8**] Apart from this, such a body could contact the patients, even interviewing them regarding their own cases. Patients should also be educated and encouraged to speak publicly regarding their own experiences.

This way, instead of rejecting important homeopathic case studies, in the name of a dry intellectualism and conservatism, homeopathy journals (including alternative and complementary journals) could become lively and interesting: initiating debates and discussions on real issues of therapeutics in medicine.

In the old homeopathy journals, we observe many such cases, and we know that by the turn of the 20th century, homeopathy was the most popular form of medicine, taught in more than one hundred homeopathic colleges in the USA. [**9**,**10**] I believe that this treatment’s popularity was due primarily to the publication of such cured cases and the discussions that followed.

Our own “Evidence Based Medicine” lies in the multitude of chronic cases treated with homeopathy that we can present to the world and on the better quality of life that such cures offer.
